# Total Tumor Volume on ^18^F-PSMA-1007 PET as Additional Imaging Biomarker in mCRPC Patients Undergoing PSMA-Targeted Alpha Therapy with ^225^Ac-PSMA-I&T

**DOI:** 10.3390/biomedicines10050946

**Published:** 2022-04-20

**Authors:** Lena M. Unterrainer, Leonie Beyer, Mathias J. Zacherl, Franz J. Gildehaus, Andrei Todica, Sophie C. Kunte, Adrien Holzgreve, Gabriel T. Sheikh, Annika Herlemann, Jozefina Casuscelli, Matthias Brendel, Nathalie L. Albert, Vera Wenter, Nina-Sophie Schmidt-Hegemann, Wolfgang G. Kunz, Clemens C. Cyran, Jens Ricke, Christian G. Stief, Peter Bartenstein, Harun Ilhan, Marcus Unterrainer

**Affiliations:** 1 Department of Nuclear Medicine, University Hospital, Ludwig Maximilian University of Munich (LMU Munich), 81377 Munich, Germany; leonie.beyer@med.uni-muenchen.de (L.B.); mathias.zacherl@med.uni-muenchen.de (M.J.Z.); franz.gildehaus@med.uni-muenchen.de (F.J.G.); andrei.todica@med.uni-muenchen.de (A.T.); sophie.kunte@med.uni-muenchen.de (S.C.K.); adrien.holzgreve@med.uni-muenchen.de (A.H.); gabriel.sheikh@med.uni-muenchen.de (G.T.S.); matthias.brendel@med.uni-muenchen.de (M.B.); nathalie.albert@med.uni-muenchen.de (N.L.A.); vera.wenter@med.uni-muenchen.de (V.W.); peter.bartenstein@med.uni-muenchen.de (P.B.); harun.ilhan@med.uni-muenchen.de (H.I.); 2 Department of Urology, University Hospital, Ludwig Maximilian University of Munich (LMU Munich), 81377 Munich, Germany; annika.herlemann@med.uni-muenchen.de (A.H.); jozefina.casuscelli@med.uni-muenchen.de (J.C.); christian.stief@med.uni-muenchen.de (C.G.S.); 3 Department of Radiation Oncology, University Hospital, Ludwig Maximilian University of Munich (LMU Munich), 81377 Munich, Germany; nina-sophie.hegemann@med.uni-muenchen.de; 4 Department of Radiology, University Hospital, Ludwig Maximilian University of Munich (LMU Munich), 81377 Munich, Germany; wolfgang.kunz@med.uni-muenchen.de (W.G.K.); clemens.cyran@med.uni-muenchen.de (C.C.C.); jens.ricke@med.uni-muenchen.de (J.R.); marcus.unterrainer@med.uni-muenchen.de (M.U.)

**Keywords:** PSMA PET/CT, total tumor volume, ^225^Ac-PSMA, mCRPC

## Abstract

Background: PSMA-based alpha therapy using ^225^Ac-PSMA-I&T provides treatment for metastatic castration-resistant prostate cancer (mCRPC), even after the failure of ^177^Lu-PSMA radioligand therapy (RLT). In clinical routine, the total tumor volume (TTV) on PSMA PET impacts therapy outcomes and plays an increasing role in mCRPC patients. Hence, we aimed to assess TTV and its changes during ^225^Ac-PSMA-I&T RLT. Methods: mCRPC patients undergoing RLT with ^225^Ac-PSMA-I&T with available ^18^F-PSMA-1007 PET/CT prior to therapy initiation were included. TTV was assessed in all patients using established cut-off values. Image derived, clinical and biochemistry parameters (PSA, LDH, AP, pain score) were analyzed prior to and after two cycles of ^225^Ac-PSMA. Changes in TTV and further parameters were directly compared and then correlated with established response criteria, such as RECIST 1.1 or mPERCIST. Results: 13 mCRPC patients were included. The median overall survival (OS) was 10 months. Prior to ^225^Ac-PSMA RLT, there was no significant correlation between TTV with other clinical parameters (*p* > 0.05 each). Between short-term survivors (STS, <10 months OS) and long-term survivors (LTS, ≥10 months OS), TTV and PSA were comparable (*p* = 0.592 & *p* = 0.286, respectively), whereas AP was significantly lower in the LTS (*p* = 0.029). A total of 7/13 patients completed two cycles and underwent a follow-up ^18^F-PSMA-1007 PET/CT. Among these patients, there was a significant decrease in TTV (median 835 vs. 201 mL, *p* = 0.028) and PSA (median 687 ng/dL vs. 178 ng/dL, *p* = 0.018) after two cycles of ^225^Ac-PSMA RLT. Here, percentage changes of TTV after two cycles showed no direct correlation to all other clinical parameters (*p* > 0.05 each). In two patients, new PET-avid lesions were detected on ^18^F-PSMA-1007 PET/CT. However, TTV and PSA were decreasing or stable. Conclusion: PET-derived assessment of TTV is an easily applicable imaging biomarker independent of other established parameters prior to ^225^Ac-PSMA RLT in these preliminary follow-up data. Even after the failure of ^177^Lu-PSMA, patients with extensive TTV seem to profit from RLT. All but one patient who was eligible for ≥2 cycles of ^225^Ac-PSMA-RLT demonstrated drastic TTV decreases without direct correlation to other biomarkers, such as serum PSA changes. Changes in TTV might hence improve the response assessment compared to standard classifiers by reflecting the current tumor load independent of the occurrence of new lesions.

## 1. Introduction

Metastatic castration-resistant prostate cancer (mCRPC) is an incurable and clinically challenging condition in prostate cancer (PC) patients, despite the availability of several approved therapy options, including second-generation antiandrogen therapy, taxane-based chemotherapy and ^223^Ra [1,2,3,4,5].

PSMA overexpression in prostate cancer represents a target for theranostic approaches in mCRPC patients. Especially, radioligand therapy (RLT) with beta-emitting ^177^Lutetium (^177^Lu)-PSMA ligands has already shown promising experiences. The first Phase III trial (VISION trial) confirmed a significantly improved progression-free and overall survival in late-stage prostate cancer patients compared to standard of care alone [6,7,8,9], whilst preserving the quality of life. Recently, PSMA-targeting alpha-particle RLT using ^225^Ac-PSMA-617 was introduced as a novel treatment option in mCRPC after exhaustion of all available treatment options, including ^177^Lu-PSMA RLT. Thus far, ^225^Ac-PSMA-617 and ^225^Ac-PSMA-I&T were evaluated in some preclinical and retrospective clinical studies [1,10,11,12,13]. In these studies, a promising antitumor activity was observed, even after exhaustion of ^177^Lu-PSMA RLT [1,10,11,14,15], keeping in mind a potential difference in tumor dose between I&T and the -617 compound [16].

In the light of PSMA-based RLT, the total tumor volume (TTV), as derived from PSMA PET/CT, has shown to be a feasible tool for evaluation of treatment response [17], as also recently underlined in a consensus statement by the EANM [18]. However, no data regarding TTV in the light of alpha therapy using ^225^Ac-PSMA-617, as well as for ^225^Ac-PSMA-I&T, are available. Hence, we assessed TTV prior to ^225^Ac-PSMA-I&T and after two cycles in comparison to other clinical and image-derived parameters and compared the TTV changes to chemical responses and further imaging-based response criteria, such as RECIST 1.1 and modified PERCIST (mPERCIST).

## 2. Materials and Methods

### 2.1. Inclusion Criteria

This retrospective analysis was approved by the institutional ethics committee of the LMU Munich (# 21-0053). Criteria for inclusion were: (1) patients with mCRPC, (2) available baseline ^18^F-PSMA-1007 PET/CT and (3) an intention to undergo a therapy regimen of baseline PET/CT, 2 cycles of ^225^Ac-PSMA-I&T and a follow-up PET/CT.

The included patients were either not eligible for or rejected other approved therapy options, or no other approved treatment options were available. The indication for RLT with ^225^Ac-PSMA-I&T was recommended by the local interdisciplinary uro-oncology conference. For more specifications, please see the flowchart in Scheme S1.

### 2.2. ^225^Ac-PSMA-I&T Radiopharmaceutical and Treatment Protocol

All patients gave written informed consent for RLT therapy, which was performed in accordance with the German Medical Products Act (AMG) §13(2b) and the updated declaration of Helsinki, paragraph 37 (Unproven Interventions in Clinical Practice). All patients were informed about the experimental nature of this therapy, as well as the potential risks and side effects. PSMA-I&T was obtained from Scintomics/ATT GmbH, Fürstenfeldbruck, Germany; ^225^Ac was obtained from ITM Medical Isotopes GmbH, Munich, Germany. For further manufacturing details, please see [1]. A 100-kBq dose of ^225^Ac-PSMA-I&T/kilogram of body weight was administered as a freehand injection over 30 s as recently described [1,10]. According to data on ^225^Ac-PSMA-617 [10], a therapy activity of 100 kBq per kilogram of body weight represents the maximum tolerable dose and activities of 150 and 200 kBq per kilogram of body weight are dose-limiting for the development of xeropthalmia and xerostomia. Patients received cool packs 30 min before and up to 4 h after injection of ^225^Ac-PSMA-I&T to cool the salivary glands for perfusion reduction. Furthermore, oral prednisone (50 mg) was administered every day; oral ondansetron (4 mg) was administered on the day of therapy. Moreover, 2 L of 0.9% intravenous NaCl was infused on the day of therapy. Inpatient stay was at least 48 h post-therapy in accordance with the German radiation protection regulations. For further details on this protocol, see also [1].

### 2.3. ^18^F-PSMA-1007 Radiopharmaceutical and Imaging Protocol

All patients gave written informed consent to undergo ^18^F-PSMA-1007 PET/CT. A median activity of 246 MBq (209–294 MBq) ^18^F-PSMA-1007 was injected intravenously in line with previously reported radiosynthesis and administration procedures [19]. Additionally, the patients were simultaneously premedicated with furosemide (20 mg) if no contraindication was given [20]. The radiopharmaceutical was used on an individual patient basis according to the German Pharmaceuticals Act §13(2b). PET was performed from the skull base to the mid-thigh using a Biograph 64 PET/CT scanner or a Biograph mCT scanner (Siemens Healthineers, Erlangen, Germany) 60 min after tracer injection PET/CT, and included a diagnostic, contrast-enhanced CT scan in a portal-venous phase (Imeron 350; 1.5 mL/kg body weight; Bracco Imaging, Milano, Italy). PET was acquired with 2.5 min per bed position and reconstructed iteratively using TrueX (three iterations, 21 subsets) with Gaussian post-reconstruction smoothing (2 mm full width at half-maximum).

### 2.4. Blood Values and Pain Score

Alkaline phosphatase (AP), lactate dehydrogenase (LDH), prostate-specific antigen (PSA) and the pain score were included in the laboratory testing and the medical history, respectively, on the day of the respective ^18^F-PSMA-1007 PET/CT before or before and after 2 further cycles of ^225^Ac-PSMA-I&T (every 2–3 months after 2 cycles of ^225^Ac PSMA-I&T).

### 2.5. Image-Based Therapy Response Assessment

Image-based treatment response was separately assessed on ^18^F-PSMA-1007 PET and CT datasets. For ^18^F-PSMA-1007 PET analysis, images were analyzed independently by two experienced nuclear medicine physicians (MU, HI) on a dedicated workstation (Hermes Hybrid 3D Viewer, Hermes Medical Solutions, Stockholm, Sweden).

#### 2.5.1. Modified PERCIST on ^18^F-PSMA-1007 PET

Transaxial PET slices were used for image analysis as described previously [21]. Five organ systems were included on a per-patient basis comprising lymph nodes, bone, affected kidney/kidney bed, and other visceral metastatic sites. Any focal uptake of ^18^F-PSMA-1007 higher than the surrounding background not associated with physiological uptake was considered suspicious for malignancy. For each organ system, the two lesions with the highest uptake were analyzed on the PET prior to therapy initiation. For quantitative analysis, the slice with the maximum ^18^F-PSMA-1007 uptake was identified using an isocontour volume of interest (VOI), including all voxels above 99% of the maximum covering the whole lesion volume. In a second step, a spherical VOI with a diameter of 1.5 cm was placed over the tumor lesion, centering in the slice with the maximum ^18^F-PSMA-1007 uptake, and the mean (SUV_mean_) and maximum standardized uptake volume (SUV_max_) were noted. Findings in the follow-up PET were compared to the baseline PET.

Posttreatment changes were interpreted according to the modified PET response criteria in solid tumors (PERCIST) 1.0 [21]. A decrease in summed SUV_mean_PERCIST_ of ≥30% was considered as a partial response (PR_PERCIST)_. The appearance of new PET-positive lesions on PET_2_ or an increase in summed SUV_mean_PERCIST_ of ≥30% was considered a progressive disease (PD_PERCIST_). An intermediate change in summed SUV_mean_PERCIST_ between −30% and +30% without new target lesions was considered a stable disease (SD_PERCIST_).

#### 2.5.2. Evaluation of Total Tumor Volume (TTV) on ^18^F-PSMA-1007 PET

Using a dedicated workstation (Affinity 3.0.1, Hermes Medical Solutions, Stockholm, Sweden) a fixed threshold of 4.0, as described previously [22], was applied, manually excluding off-target, PSMA-avid lesions. A decrease in TTV, SUV_mean_TTV_ or SUV_max_TTV_ of ≥30% was considered as PR_TTV_. The appearance of new PET-positive lesions on PET_2_ or an increase in TTV, SUV_mean_TTV_ or SUV_max_TTV_ of ≥30% was considered a progressive disease (PD_TTV_). An intermediate change in TTV, SUV_mean_TTV_ or SUV_max_TTV_ between −30% and +30% without new target lesions was considered a stable disease (SD_TTV_).

#### 2.5.3. CT (RECIST 1.1)

For the evaluation of CT datasets, a response assessment was performed by two experienced radiologists (WGK, MU) according to RECIST 1.1 using dedicated software (mint lesion^TM^, version 3.0.1, Mint Medical GmbH, Heidelberg, Germany) [21,23]: Target and non-target lesions were defined and measured in baseline CT prior to therapy initiation. In the corresponding follow-up CT examination, target lesions were located and manually measured. The disappearance of all lesions was considered a complete response (CR_CT_), a decrease in summed diameters of ≥30% was defined as a partial response (PR_CT_). The appearance of a new target lesion on CT_2_ or an increase in the summed diameters of ≥20% with an absolute increase of at least 5 mm was defined as progressive disease (PD_CT_). An intermediate change in the summed diameter between −30% and +20% without the appearance of a new target lesion was considered a stable disease (SD_CT_).

### 2.6. Follow-Up

Follow-up-time was calculated from the date of the ^18^F-PSMA-1007 PET/CT prior to the first cycle of ^225^Ac PSMA-I&T to the date of death or date of loss to follow-up.

### 2.7. Statistical Analysis

Statistical analyses were performed with IBM SPSS^®^ Statistics (version 25; SPSS, Chicago, IL, USA). Descriptive statistics are displayed as median (range). Relative changes during therapy are displayed as percentage differences. Correlation analyses between different PET parameters and/or other clinical parameters were evaluated using Spearman’s correlation coefficient after testing for normal distribution, as determined by the Shapiro–Wilk test. Group comparisons were performed using a non-parametric paired Wilcoxon test and a non-parametric Fisher’s exact test. Patients were divided into long-term and short-term survivors by using median split dichotomization. Statistical significance was defined as a two-sided *p*-value < 0.05.

## 3. Results

### 3.1. Patient Characteristics at Baseline Time Point

Thirteen patients with mCRPC and an ^18^F-PSMA-1007 PET/CT prior to RLT received at least one cycle of ^225^Ac-PSMA-I&T: A total of 31 cycles of ^225^Ac-PSMA-I&T were applied (median dose of 7.6 MBq, range 6.0–8.5). Four patients received one cycle, five patients received two cycles, three patients received four cycles and one patient received five cycles. Seven patients completed two cycles with clinical follow-up, including ^18^F-PSMA-1007 PET/CT. Detailed patient characteristics, including blood values, metastatic spread and pain score prior to therapy are provided in Table 1. The median follow-up time was 10.0 months.

### 3.2. Correlation of TTV and Further Pretherapeutic Parameters

**Figure 1:**
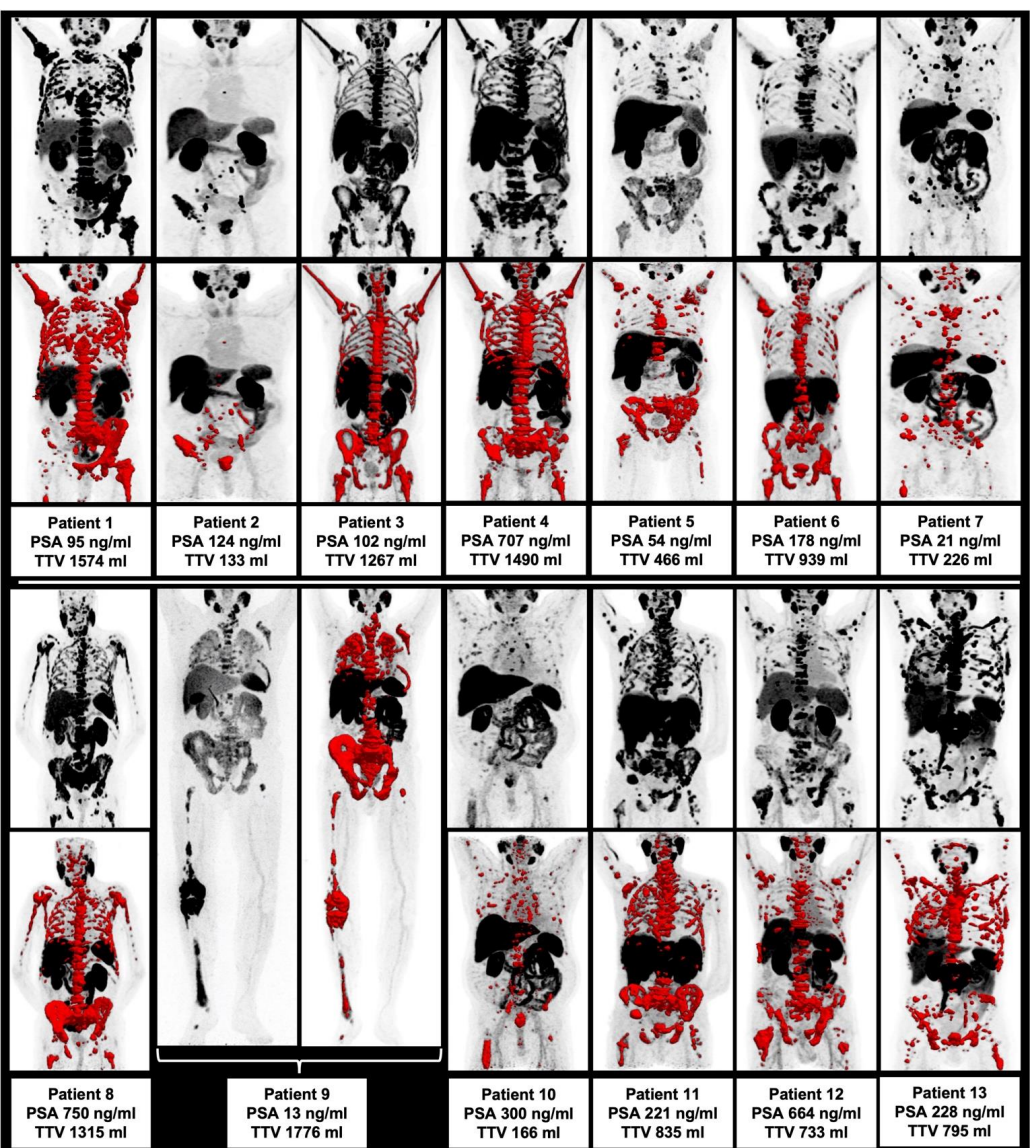
Overview of all included patients with maximum intensity projection (MIP) and included TTV (red).

Prior to the first cycles of ^225^Ac-PSMA-I&T, there was no significant correlation of TTV with other image and clinically derived parameters (*p* > 0.05 each), i.e., with AP, LDH, PSA, pain score and SUV_mean/_SUV_max_, as derived from the TTV assessment. The highest correlation was observed correlating PSA/AP and LDH/AP without reaching the level of significance (i.e., *p* = 0.076 and *p* = 0.059, respectively). Hence, there was no statistically significant intercorrelation of the assessed parameters prior to ^225^Ac-PSMA-I&T noted. Please see Table 2. All patients at baseline prior to RLT are displayed in Figure 1.

### 3.3. Correlation of TTV and Pretherapeutic Parameters with Clinical Outcome

The median OS was 10.0 months (95% CI 6.2–15.2 months). The survival rates were: 4 months survival rate 84.6 % (11/13), 8 months survival rate 53.8 % (7/13), 12 months survival rate 33.3% (4/12), 18 months survival rate 25.0% (3/12) and 24 months survival rate 8.3% (1/12). Median split dichotomization using the cut-off of 10 months was used to divide patients into short-term survivors (STS, <10 months OS) and long-term survivors (LTS, ≥10 months OS). The PET-derived TTV was comparable between STS and LTS (median 815 vs. 939 mL, *p* = 0.592) at baseline; the same was notable regarding PSA (median 224.5 vs. 124 ng/dL, *p* = 0.286). AP was significantly lower in LTS compared to STS (median 412.5 vs. 98 U/L, *p* = 0.029). All other clinical and image-derived parameters were comparable between STS and LTS patients (*p* > 0.05 each). Please see the overview in Table 3.

### 3.4. Changes of Clinical and Image-Derived Parameters during ^225^Ac-PSMA-I&T Treatment

Among 13 patients who started ^225^Ac-PSMA-I&T treatment, 7/13 (53.8%) completed a treatment regimen of two cycles and clinical follow-up using ^18^F-PSMA-1007 PET/CT. Among these patients, there was a significant decrease of TTV at follow-up compared to the baseline (median 835 vs. 201 mL, *p* = 0.028) with a median decrease of 62.3%. A total of 5/7 (71.4%) patients had a TTV decrease >50%, 6/7 (85.7%) patients had a TTV decrease >30%.

Moreover, there was a significant decrease of PSA after two cycles of ^225^Ac-PSMA-I&T (median 178 vs. 686.6 ng/dL, *p* = 0.018) with a median decrease of 32.8%. A total of 1/7 (14.3%) patients had a PSA decrease >50%, 4/7 (57.1%) patients had a PSA decrease >30%.

**Figure 2:**
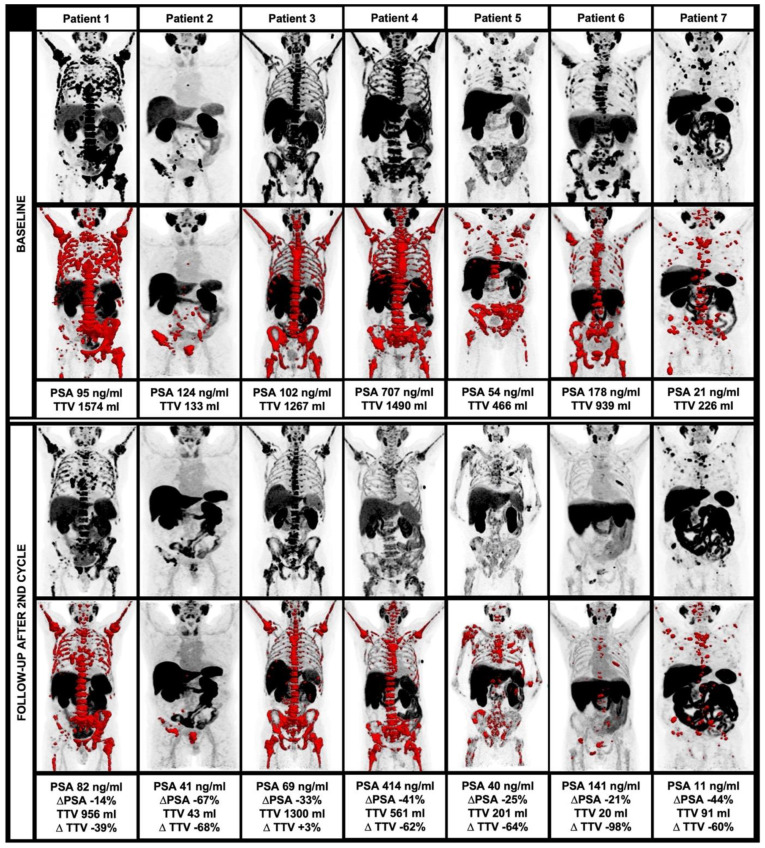
Course of TTV and clinical parameters during alpha therapy, including MIP and TTV (red) at baseline (**upper row**) and at follow-up after the second therapy cycle (**lower row**).

SUV_mean_, as derived within the TTV also significantly decreased after two cycles of ^225^Ac-PSMA-I&T (median 9.5 vs. 7.1, *p* = 0.018), whereas all other parameters showed no significant changes in the follow-up (*p* > 0.05 each). Overall, there was a trend toward a higher percentage decrease of TTV compared to PSA without reaching the level of significance (median 62.3% vs. 32.8%, *p* = 0.128). For further information, please see Table 4. The changes in TTV are displayed in Figure 2.

### 3.5. Intercorrelation of Clinical and Imaged Derived Changes after ^225^Ac-PSMA-I&T Treatment

Again, percentage changes of TTV after two cycles of ^225^Ac-PSMA-I&T showed no significant correlation to all other parameters (*p* > 0.05 each). Moreover, percentage changes of all other parameters after treatment (i.e., PSA, AD, LDH, SUV_mean_, SUV_max_ and pain score) showed no significant correlation; hence, changes in clinical and image-derived parameters after two cycles of RLT showed no statistically significant intercorrelation. See also Table 5.

### 3.6. Changes of TTV in Comparison to Further Response Criteria

Overall, there was a median percentage decrease of TTV of −62.3% (range −97.8%–+2.6%) resulting in a response classification of six patients with PR and one with SD that showed stable, non-decreasing TTV (i.e., TTV +2.6%).

The response assessment using SUV_mean_ comprised five patients with SD and only two patients with PR, whereas the response assessment, using SUV_max_ resulted in diverging classifications of five patients with PR, but also two patients with PD due to increasing uptake intensity despite significantly decreasing TTV (i.e., −62.4%/−64.5%) and PSA (−41.4%/−25.2%).

Applying an mPERCIST approach to the response assessment to ^225^Ac-PSMA-I&T RLT, five patients were rated PR, but two patients were rated PD due to new PSMA-avid bone lesions with only a partial morphological correlate on CT imaging; in these two patients, TTV was decreasing and stable (−64.5%/+2.6%) and PSA was decreasing (−32.8%/−25.2%).

Using RECIST 1.1, three patients were classified as Non-CR/Non-PD due to missing target lesions at baseline. Three patients were classified as SD, and one patient as PD due to new PSMA-avid lesions with a morphological correlate on CT imaging. Detailed information regarding diverging response classifiers can be found in Table 6.

### 3.7. Occurrence of New Lesions in Correlation to TTV and Further Parameters

In two patients (patients 3 and 5), new lesions were noted on ^18^F-PSMA-1007 PET/CT imaging after two cycles of RLT; in one case, new lymph node metastases with high PSMA-expression, but also pathological enlargement on CT were present, so that there were PD ratings using RECIST (new lesions), mPERCIST (new PSMA-avid lesions) and using SUV_max_ derived from the TTV (>30% increase of SUV_max_). However, there was a decrease in TTV of 64.5% despite these new lesions. Moreover, PSA was decreasing by 25.2%. Moreover, new bone lesions with high PSMA-expression were noted, predominantly without distinct CT correlate.

**Figure 3:**
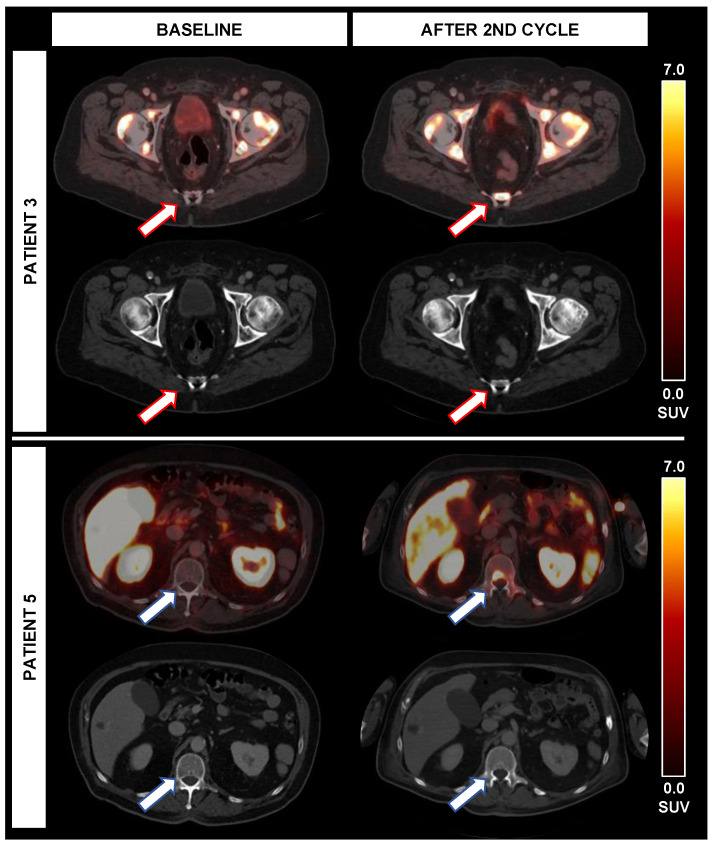
Occurrence of new PSMA-avid lesions during alpha therapy. While patient 3 experienced new PSMA-avid lesions (red arrows) without correlate on CT, the TTV (+3%) was stable and PSA (−33%) was decreasing after 2 cycles. In patient 5, new bone lesions (blue arrow) and lymph nodes with PSMA-expression were noted after the second cycle; however, both TTV (−65%) and PSA (−25%) were decreasing despite new lesions.

In the second case, new PSMA-expressing bone lesions were noted, also predominantly without distinct CT correlate, partially with expanding sclerotic areas. This led to diverging response classifications using RECIST (Non-CR/Non-PD, only sclerosis without new targetable lesions), mPERCIST (new PSMA-avid lesions) but also decreasing SUV_max_ by 81.6%. However, despite these new bone lesions, TTV was stable (+2.6% TTV) and PSA was decreasing (−32.8%). The new bone lesions of these cases are displayed in Figure 3.

## 4. Discussion

PET-derived TTV is considered to play an increasing role in response assessment in mCRPC patients [18,24,25]. To the best of our knowledge, there is no study evaluating baseline or interim TTV in comparison to the clinical course of RLT with ^225^Ac-PSMA-I&T in mCRPC patients.

Firstly, the assessment of the TTV was easily applicable and highly feasible in all included patients using the same PET ligand ^18^F-PSMA-1007. As a first step, we assessed whether TTV demonstrates a significant intercorrelation with other established clinical and image-derived parameters. Our data did not find any significant intercorrelation, especially between TTV and PSA. This is an important finding, as PSA is usually considered the clinical gold standard for biochemical monitoring in mCRPC patients [21]. Hence, we can assume that TTV is a potential independent imaging biomarker without intercorrelation to other clinical parameters prior to RLT with ^225^Ac-PSMA.

Overall, the median OS was 10 months, which is in line with current studies and meta-analyses [11,14,26]. Interestingly, the two patients with the longest survival had the lowest pre-treatment TTV, suggesting a possible predictive role for TTV.

To assess potential negative prognosticators prior to RLT with ^225^Ac-PSMA, we performed a median split dichotomization of the current patient group with a cut-off of 10 months, as it represents the current median OS. The only parameter that was significantly different in STS and LTS was AP; AP has been shown to be of interest as a prognosticator in mCRPR patients undergoing different lines of therapy [27,28], especially in the case of undergoing RLT with ^177^Lu-PSMA, as presented in the German multicenter study [29] and in further analyses of data from the TheraP trial [30]. However, TTV was comparable between STS and LTS patients, allowing the hypothesis that even patients with an extensive tumor load in terms of TTV might profit from RLT with ^225^Ac-PSMA-I&T and should not be excluded from RLT in the first place.

Among 13 patients intended to treat, 7 patients completed a therapy regimen of two cycles and clinical follow-up, including ^18^F-PSMA-1007 PET/CT. After therapy initiation, six patients were not able to undergo further therapy cycles due to decreasing clinical conditions or declined further treatments/changed to best supportive care. After two cycles of ^225^Ac-PSMA, there was a significant decline in TTV with 71% of patients with >50% and 86% of patients with >30% TTV decrease. The percentage decrease of TTV was even higher than the percentage decrease of PSA, with only 15% and 57% of patients showing a decrease of 50% and 30%, respectively, which is in line with the current literature [11]. A comparable decrease could be observed regarding SUV_mean_ as derived from TTV. However, the percentage differences (baseline vs. follow-up after two cycles ^225^Ac-PSMA) of all parameters included, again showed no significant intercorrelation.

Although all those patients eligible for clinical follow-up experienced a PSA decline, all but one patient experienced a TTV decrease; the percentage decrease of both parameters was not directly correlated with each other (*r* = 0.107, *p* = 0.819). Moreover, all other parameters, including AP, were not intercorrelated. This missing intercorrelation underlines that TTV on PET might potentially serve as an additional imaging biomarker independent of patients’ PSA.

As proposed by recent studies [31,32], we applied a response classifier based on TTV; here all but one patient demonstrated PR and one patient showed SD, reflecting the overwhelmingly decreasing TTV after two cycles. Nonetheless, the response on TTV was highly divergent in direct comparison to other criteria, especially since other PET-based classification systems are primarily based on uptake intensity, such as the response based on SUV_mean_ and SUV_max_. Overall, significant decreases in TTV can be observed whilst still presenting highly PSMA-avid lesions. Keeping in mind the palliative setting and very low likelihood of a “complete metabolic response” in these extensively pre-treated patients, it seems clinically justified to assume that the mere assessment of uptake intensity does not reflect the objective tumor burden and its changes during certain treatments.

Especially, responses based on mere morphological imaging, as defined by the RECIST 1.1 criteria, does not seem suitable at all in this patient population with partially extensive tumor loads and, particularly, with a high proportion of bone-dominant tumor load. Given the already known and extensively discussed limitations of standard criteria such as RECIST 1.1, especially for the assessment of bone lesions on CT imaging (e.g., no soft tissue involvement, no osseous target lesions, new sclerosis, etc.) [23,33,34,35], novel aspects of molecular response assessment, such as TTV, are desirable to overcome these limitations in mCRPC patients [18]. Of note, the only criteria that showed a concordant trend toward a common response were PSA and TTV; however, the amount of their decrease was not correlated with each other at all, so a certain independency, despite a common trend towards decrease, can be postulated.

Of note, the phenomenon of new lesions in light of PSMA-directed RLT remains a matter of debate, as recently stated in the EANM consensus statement concerning the response assessment criteria in prostate cancer [18]. In the current analysis, we observed two patients who experienced new lesions after two cycles of ^225^Ac-PSMA RLT. Accordingly, these two cases were classified as PD in several response criteria, such as RECIST 1.1 or mPERCIST, also depending on the morphological appearance and localization; however, both PSA and TTV were decreasing or were at least stable in these patients. These two cases illustrate the area of tension of classical perception in the case of newly occurring lesions and the intuitive link to disease progression. Nevertheless, patients undergoing PSMA-directed RLT are usually presenting with extensive tumor load, and in a generally palliative setting, with limited clinical relevance of a single newly occurring lesion, there is justified debate if this scenario should be formally classified as disease progression. Particularly, it seems quite intuitive to set the occurrence of new lesions in relation to the remaining stable or even decreasing tumor load, as assessed by the TTV on PSMA PET.

**Figure 4:**
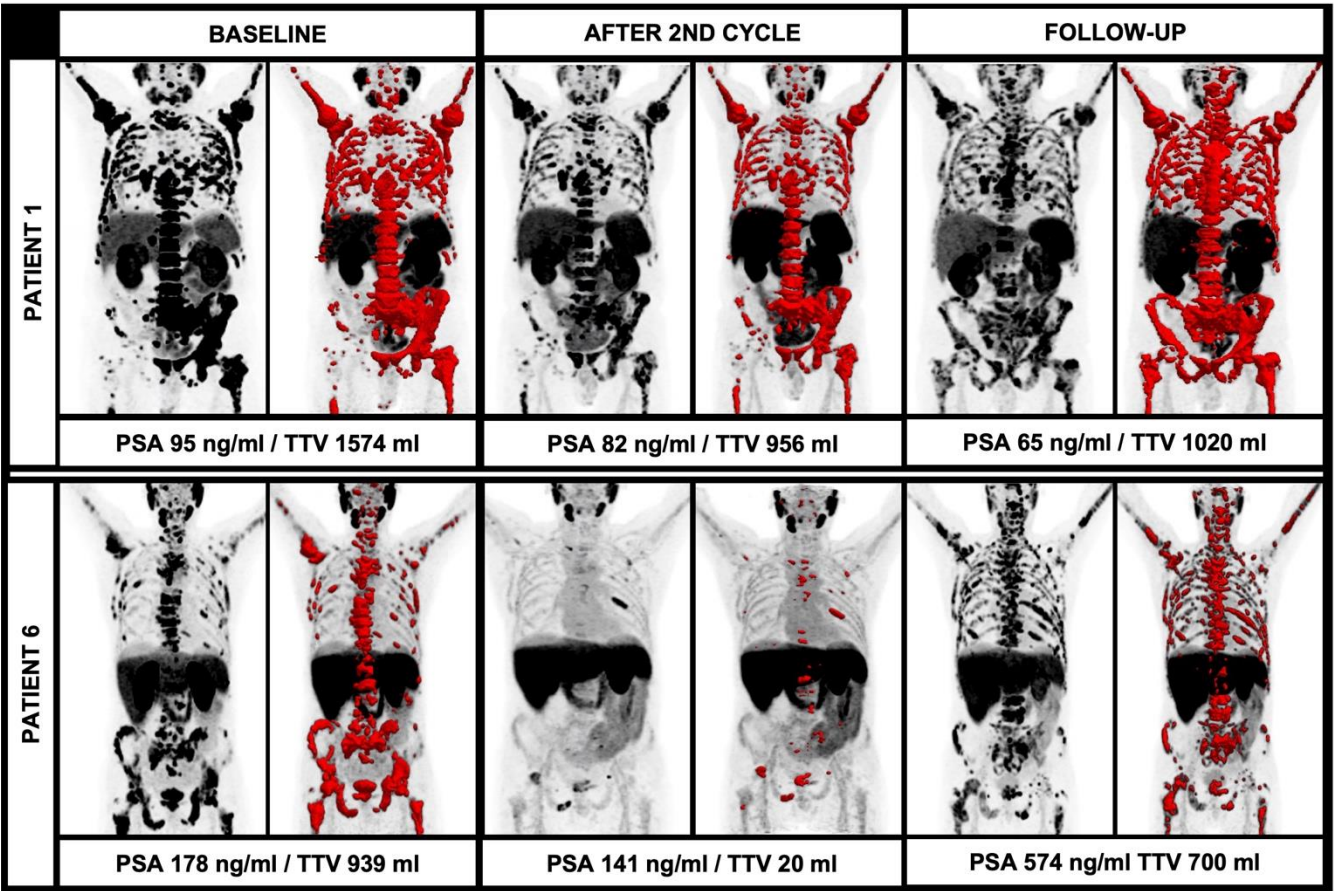
Diverging course of TTV with newly occurring lesions during alpha therapy. While patient 1 had a stable TTV and decreasing PSA during therapy despite new PSMA-avid lesions, patient 6 had new PSMA-avid lesions with an extensive increase of TTV and PSA.

In this regard, the current EANM consensus statement discusses that an increase of at least 30% of the TTV and/or the appearance of two new lesions should be rated as disease progression. However, the mere number of new lesions is not explicitly assessable in the light of the extensive tumor load in most end-stage patients undergoing RLT due to the occurrence of vast confluent bone sclerosis and partially coalescing, PSMA-avid tumor lesions. The theory of disease progression in relation to the TTV is underlined by the current data: In the further follow-up, there were also patients with multiple new lesions, but diverging TTV during further RLT with ^225^Ac-PSMA-I&T; patient 1 showed several new lesions after four cycles or RLT, but only a moderate increase of TTV (956 mL to 1020 mL, +7%) and a decreasing PSA (82 ng/mL to 65 mg/dL, −21%). On the other hand, there were cases with new lesions in the further clinical workup with an extensive increase of the TTV and PSA, e.g., patient 6; after the initial TTV decrease, there were numerous new lesions accompanied by an extensive increase of the TTV (20 mL to 700 mL, +3500%) and a rising PSA (141 ng/mL to 574 ng/mL, +407%). These cases illustrate the different impacts of new lesions in light of the overall TTV (please see Figure 4). Given the paucity of studies evaluating alpha therapy using ^225^Ac-PSMA ligands, further studies with larger patient cohorts have to address the issue of defining disease progression or even therapy failure. Especially, clear absolute or relative changes of TTV in correlation with other parameters have to be further elucidated to improve response assessment in this patient group with partially large tumor load and also extensive pre-treatments.

Some limitations of our study must be addressed: firstly, limitations that arise from the retrospective design. Moreover, the low number of patients included hampers in-depth statistical analyses; however, all patients underwent the same PET/CT scan with the same ligand ^18^F-PSMA-1007, which enables direct comparability. Nonetheless, we want to share our preliminary data as it might have an impact on how to evaluate response under ^225^Ac-PSMA ligand therapy, particularly in future clinical trials.

Moreover, more standardization regarding treatment regimens, follow-up and image controls are needed to improve the comparability of results and to foster multicenter comparisons of data regarding ^225^Ac-PSMA-I&T. In this light, the survival analysis remains on a superficial level; especially, the prognostic values of TTV changes cannot be solved in this rather small sample size; this issue must be addressed by prospective study designs and multicenter data pooling, given the low number of sites with ^225^Ac availability, as of today.

Moreover, given the extensive pre-treatments of the current cohort, a certain biological tumor selection or even de-differentiation has probably taken place and has to be considered. However, other imaging biomarkers associated with de-differentiation reflected by the loss of PSMA-expression and increasing FDG-avidity were not assessed in this study, and are however part of ongoing prospective studies assessing ^177^Lu-PSMA RLT [36]. Therefore, the prognostic value of further de-differentiated tumor clones as reflected by ^18^F-FDG PET and its potential as “gate-keeper” prior to RLT with ^225^Ac-PSMA and its correlation to the TTV on PSMA PET must be assessed further.

## 5. Conclusions

PET-derived assessment of TTV is an easily applicable imaging biomarker that seems independent of other established clinical parameters prior to ^225^Ac-PSMA RLT in this pilot study. Even patients with extensive TTV seem to profit from RLT. Most patients eligible for ≥2 cycles ^225^Ac-PSMA-RLT show drastic TTV decreases without direct correlation to other biomarkers, such as serum PSA changes. Evaluating changes in TTV might hence improve response assessment compared to standard response classifiers, such as RECIST 1.1 and mPERCIST, by reflecting the current tumor load independently from the occurrence of new lesions.

## Figures and Tables

**Table 1:** Baseline characteristics prior to ^225^Ac-PSMA-I&T.

Parameters	Value [Median, Range]
Age [years]	74.8 (65.8–80.9)
PSA [ng/mL]	178.0 (13.4–750.0)
AP [U/L]	228.0 (68.0–695.0) (normal range 40.0–130.0)
LDH [U/L]	344.0 (150.0–1457.0) (normal range <249.0)
Pain Score	5.0 (0.0–8.0)
TTV [mL]	835.0 (133.0–1776.0)
SUV_mean_	9.5 (6.6–15.8)
SUV_max_	42.8 (17.5–175.9)
Sites of tumor spread	
Bone	13/13 (100.0%)
Lymph node	8/13 (61.5%)
Visceral metastases	4/13 (30.8%)
Local tumor	7/13 (53.8%)
Prior therapies	
Prostatectomy	4/13 (30.7%)
Radiotherapy	7/13 (53.8%)
ADT	13/13 (100.0%)
Docetaxel	9/13 (69.2%)
^177^Lu-PSMA-RLT	10/13 (76.9%)
^223^Ra-dichlorode	1/13 (7.7%)

**Table 2:** Correlation of clinical and image-derived parameters prior to the first cycle of ^225^Ac-PSMA-I&T.

	PSA	AP	TTV	SUV_mean_	SUV_max_	LDH
AP	*r* = 0.509 (*p* = 0.076)	-	-	-	-	-
TTV	*r* = −0.022 (*p* = 0.943)	*r* = 0.036 (*p* = 0.908)	-	-	-	-
SUV_mean_	*r* = −0.324 (*p* = 0.280)	*r* = −0.281 (*p* = 0.353)	*r* = 0.181 (*p* = 0.553)	-	-	-
SUV_max_	*r* = −0.341 (*p* = 0.255)	*r* = −0.426 (*p* = 0.146)	*r* = 0.143 (*p* = 0.642)	*r* = 0.725 (*p* = 0.005)	-	-
LDH	*r* = 0.148 (*p* = 0.629)	*r* = 0.536 (*p* = 0.059)	*r* = 0.346 (*p* = 0.247)	*r* = −0.429 (*p* = 0.144)	*r* = −0.319 (*p* = 0.289)	-
Pain Score	*r* = −0.031 (*p* = 0.920)	*r* = 0.070 (*p* = 0.820)	*r* = 0.020 (*p* = 0.949)	*r* = −0.205 (*p* = 0.503)	*r* = −0.437 (*p* = 0.135)	*r* = 0.014 (*p* = 0.964)

**Table 3:** Comparison of baseline characteristics between short-term survivors (STS; overall survival (<10 months) compared to patients with an overall survival ≥10 months (long-term survivors, LTS) as defined by median split dichotomization.

Parameters	STS (*n* = 6) [Median (Range)]	LTS (*n* = 7) [Median (Range)]	Significance
PSA [ng/dL]	224.5 (13.4–750.0)	124.0 (20.5–707.0)	*p* = 0.286
AP [U/L]	412.5 (155.0–695.0)	98.0 (68.0–326.0)	*p* = 0.029
LDH [U/L]	585.0 (264.0–1457.0)	274.0 (150.0–616.0)	*p* = 0.286
Pain score	5.0 (0.0–8.0)	4.0 (0.0–7.0)	*p* = 0.559
TTV [mL]	815.0 (566.0–1776.0)	939.0 (133.0–1574.0)	*p* = 0.592
SUV_mean_	9.5 (6.9–15.15)	9.2 (6.6–15.6)	*p* = 1.000
SUV_max_	36.8 (17.5–113.6)	55.0 (38.5–175.9)	*p* = 0.592

**Table 4:** Changes of image-derived and clinical parameters prior to and after two cycles of ^225^Ac-PSMA-I&T.

Parameters	Baseline [Median (Range)]	Follow-up [Median (Range)]	Δ% [Median (Range)]	Significance
PSA [ng/dL]	178.0 (13.4–750.0)	68.6 (11.4–414.0)	−32.8 (−67.3–−14.2)	* p * = 0.018
AP [U/L]	228.0 (68.0–695.0)	148.0 (61.0–487.0)	−20.5 (−30.6–165.3)	* p * = 0.176
LDH [U/L]	344.0 (150.0–1457.0)	304.0 (174.0–1124.0)	−3.5 (−29.6–126.6)	* p * = 0.866
TTV [mL]	835.0 (133.0–1776.0)	201.0 (20.3–1300.0)	−62.3 (−97.8–2.6)	* p * = 0.028
SUV_mean_	9.5 (6.6–15.8)	7.1 (6.3–9.7)	−24.1 (−42.3–−8.1)	* p * = 0.018
SUV_max_	42.8 (17.5–175.9)	32.6 (16.2–54.3)	−46.0 (−81.6–74.2)	* p * = 0.063

**Table 5:** Correlation of percental changes of clinical and image-derived parameters after two cycles of ^225^Ac-PSMA-I&T.

	Δ% PSA	Δ% AP	Δ% TTV	Δ% SUV_mean_	Δ% SUV_max_
Δ% AP	*r* = 0.071 (*p* = 0.879)	-	-	-	-
Δ% TTV	*r* = 0.107 (*p* = 0.819)	*r* = 0.000 (*p* = 1.000)	-	-	-
Δ% SUV_mean_	*r* = 0.214 (*p* = 0.645)	*r* = 0.571 (*p* = 0.180)	*r* = −0.250 (*p* = 0.589)	-	-
Δ% SUV_max_	*r* = −0.286 (*p* = 0.535)	*r* = −0.321 (*p* = 0.482)	*r* = −0.250 (*p* = 0.589)	*r* = −0.071 (*p* = 0.879)	-
Δ% LDH	*r* = −0.286 (*p* = 0.535)	*r* = −0.536 (*p* = 0.215)	*r* = 0.107 (*p* = 0.819)	*r* = 0.000 (*p* = 1.000)	*r* = 0.571 (*p* = 0.180)

**Table 6:** Comparison of different response classifiers among patients undergoing ≥2 cycles and with follow-up ^18^F-PSMA-1007 PET/CT after the second cycle.

Patient	Response TTV	Δ% TTV	Response SUV_mean_	Δ% SUV_mean_	Response SUV_max_	Δ% SUV_max_	Response mPERCIST	Δ% mPERCIST	Response RECIST	Δ% RECIST	Response RECIST	Δ% PSA	Δ% LDH	Δ% AP	OS [mo]
1	PR	−39.3%	PR	−37.5%	PR	−54.9%	PR	−36.8%	SD	+2.6%	SD	−14.2%	−3.5%	−30.6%	10
2	PR	−67.9%	SD	−24.1%	PR	−46.0%	PR	−65.3%	SD	−13.9%	SD	−67.3%	+16.0%	−10.3%	26
3	SD	+2.6%	SD	−21.6%	PR	−81.6%	PD *	n.e.	Non-CR/Non-PD	n.a.	Non-CR/Non-PD	−32.8%	−11.6%	+165.3%	18
4	PR	−62.4%	SD	−24.1%	PD	+33.6%	PR	−60.5%	Non-CR/Non-PD	n.a.	Non-CR/Non-PD	−41.4%	−23.8%	+42.9%	11 °
5	PR	−64.5%	SD	−8.1%	PD	+74.2%	PD *	n.e.	PD	n.e.	PD	−25.2%	+126.6%	+20.5%	5
6	PR	−97.8%	SD	−23.3%	PR	−62.2%	PR	−66.4%	SD	+8.9%	SD	−20.8%	−29.6%	+55.8%	11
7	PR	−59.6%	PR	−42.3%	PR	−45.2%	PR	−78.5%	Non-CR/Non-PD	n.a.	Non-CR/Non-PD	−44.4%	+22.0%	−9.2%	20

* New PSMA-avid lesion; n.e.: not evaluated by new PET-avid lesion or new lesion in RECIST 1.1 and consecutive progressive disease; n.a. not available due to Non-CR/Non-PD in RECIST 1.1; ° censored, lost to follow-up.

## Supplementary Materials

The following supporting information can be downloaded at: https://www.mdpi.com/article/10.3390/biomedicines10050946/s1, Scheme S1. Flowchart with patients’ inclusion and analysis algorithm.
