# Supplementary figures and images for: Total Tumor Volume on 18F-PSMA-1007 PET as Additional Imaging Biomarker in mCRPC Patients Undergoing PSMA-Targeted Alpha Therapy with 225Ac-PSMA-I&T
Source: Biomedicines. 2022 Apr 20;10(5):946. doi: 10.3390/biomedicines10050946 (PMC9138410; doi:10.3390/biomedicines10050946)

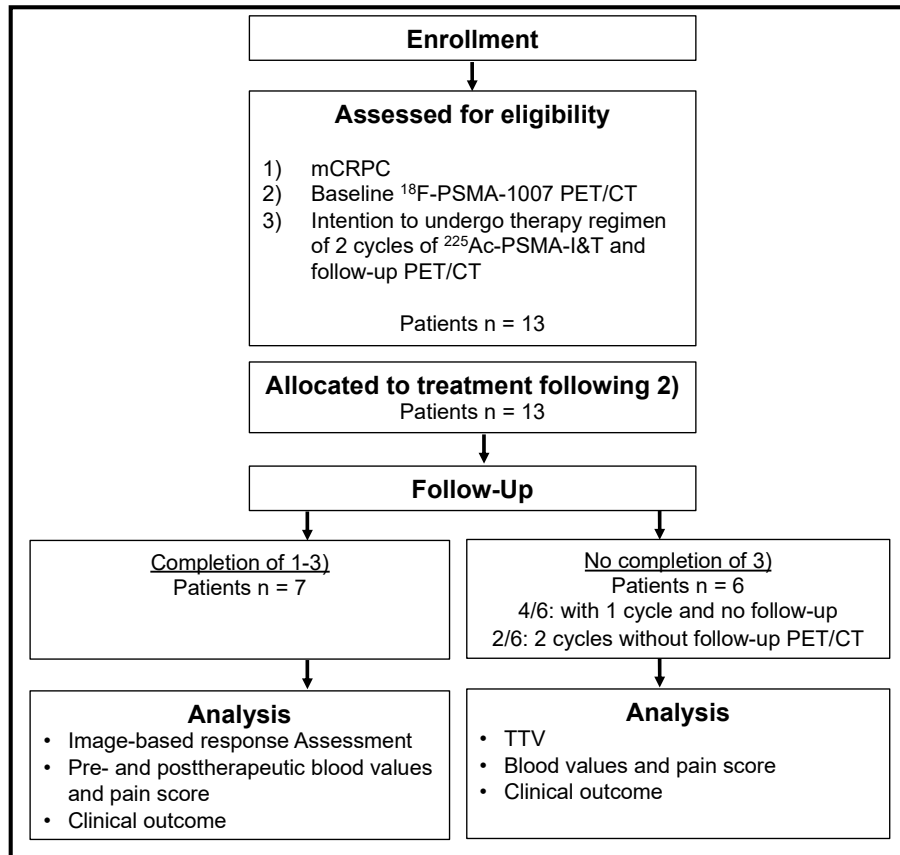

**Scheme S1.** Flowchart with patients' inclusion and analysis algorithm.

Supplement: Supplementary file 1 [file biomedicines-10-00946-s001.zip › biomedicines-1645768-supplementary.pdf]
